# Utilization of Chinese medicine for respiratory discomforts by patients with a medical history of tuberculosis in Taiwan

**DOI:** 10.1186/s12906-018-2377-4

**Published:** 2018-11-29

**Authors:** Su-Tso Yang, Yi-Rong Lin, Mei-Yao Wu, Jen-Huai Chiang, Pei-Shan Yang, Te-Chun Hsia, Hung-Rong Yen

**Affiliations:** 1 Graduate Institute of Chinese Medicine, School of Chinese Medicine, College of Chinese Medicine, China Medical University, Taichung, Taiwan; 2 Department of Radiology, China Medical University Hospital, Taichung, Taiwan; 3 Department of Chinese Medicine, China Medical University Hospital, Taichung, Taiwan; 4 Management Office for Health Data, China Medical University Hospital, Taichung, Taiwan; 5 Department of Respiratory Therapy, China Medical University, Taichung, Taiwan; 6 Department of Internal Medicine, China Medical University Hospital, Taichung, Taiwan; 7 Research Center for Traditional Chinese Medicine, Department of Medical Research, China Medical University Hospital, Taichung, Taiwan; 8 Research Center for Chinese Herbal Medicine, China Medical University, Taichung, Taiwan; 9 Chinese Medicine Research Center, China Medical University, Taichung, Taiwan; 10 Department of Biotechnology, Asia University, Taichung, Taiwan

**Keywords:** Chinese medicine, National Health Insurance Research Database, Prescription, Respiratory diseases, Tuberculosis

## Abstract

**Background:**

Tuberculosis (TB) is one of the world’s major communicable infectious diseases, and it still imposes a great health burden in developing countries. The development of drug-resistant TB during the treatment increases the treatment complexity, and the long-term pulmonary complications after completing treatment raise the epidemic health burden. This study intended to investigate the utilization of Chinese medicine (CM) for respiratory symptoms by patients with a medical history of TB in Taiwan.

**Methods:**

We analyzed a cohort of one million individuals who were randomly selected from the National Health Insurance Research Database in Taiwan. The inclusion criteria of patients (*n* = 7905) with history of TB (ICD-9-CM codes 010–018 and A02) were: (1) TB diagnosed between January 1, 1997 and December 31, 2010 (2) 18 years old or over (3) Clinical records for at least 2 months with complete demographic information (4) Record of treatment with first-line TB medication prescriptions. CM users for conditions other than respiratory discomforts (*n* = 3980) were excluded. Finally, a total of 3925 TB patients were categorized as: CM users for respiratory discomforts (*n* = 2051) and non-CM users (*n* = 1874).

**Results:**

Among the 3925 subjects, 2051 (52.25%) were CM users, and 1874 (44.753%) were non-CM users. Female patients and those who were younger (18–39 y/o) and who lived in urbanized areas relatively tended to be CM users (*p* < .0001). Most of the CM users (1944, 94.78%) received Chinese medicines. The most commonly prescribed herbal formulas and single herbs were Xiao-Qing-Long-Tang and Radix Platycodonis (Jie-Geng), respectively. The core pattern of Chinese medicines for TB patients consisted of Ma-Xing-Gan-Shi-Tang, Bulbus Fritillariae Thunbergii (Bei-Mu), Radix Platycodonis (Jie-Geng) and Semen Armeniacae (Xing-Ren).

**Conclusions:**

The use of CM is popular among patients with a medical history of TB complicated with long-term respiratory discomforts in Taiwan. Further pharmacological investigations and clinical trials are required.

## Introduction

In the twenty-first century, tuberculosis (TB) continues to be one of the world’s major health challenges. TB is a deadly communicable disease that is primarily transmitted from human to human by droplet infection, and it is a major health burden in both developed and developing countries. Approximately 9.0 million people developed TB, and 1.5 million died from the disease in 2013 [1]. Of the 9.0 million estimated cases, 56% were in Southeast Asian and Western Pacific regions [2]. China accounted for 11% of the 9.0 million estimated TB cases, and 11,528 TB cases were confirmed in Taiwan [3]. Male, elderly and immunocompromised patients, including those with HIV, patients with poorly controlled diabetes mellitus or chronic kidney disease or those receiving immunosuppressant treatments, are generally more susceptible to the disease [4]. Healthcare workers who are exposed to TB also have higher risks of being infected [5].

Due to the side effects of the drugs [6, 7], poor medication compliance and the problem of development of drug-resistant TB, global investment in TB research is needed [8]. Among the first-line anti-TB drugs, side effects of hepatotoxicity have been reported to be associated with isoniazid, rifampin and pyrazinamide; cutaneous reactions with isoniazid, pyrazinamide and ethambutol; gastrointestinal intolerance with rifampin; retrobulbar neuritis with ethambutol; and ototoxicity with streptomycin [9–11]. Long-term respiratory symptoms and impairment of pulmonary function affect patients’ quality of life after completing TB treatment. The respiratory comorbidities were identified in more than half of microbiologically cured TB patients [12]. Despite successful treatment of TB, residual impairments persist, and chronic complications or sequelaes can arise from structural or vascular alterations at disease sites [13, 14]. Many patients with chronic illness may seek complementary therapies [15], especially Chinese Medicine (CM) in Asian countries [16, 17]. CM, which originated in ancient China, is defined as comprehensive healthcare skills and practice for the maintenance of health and treatment of disease based on the beliefs of holism [18] and experiences of pattern identification/syndrome differentiation handed down from generation to generation [19]. In Taiwan, CM is important and popular among different categories of complementary therapies, and is regarded as one of the mainstream therapies with coverage of the National Health Insurance (NHI) program [17]. “CM” usually refers to the overall treatment modalities, and services of CM covered by the NHI program, which includes Chinese medicines, acupuncture, moxibustion and Chinese traumatology therapy [20]. “Chinese medicines” usually refers to herbs or herbal products which can be classified into single-herb products and herbal formulas (multi-herb products) [21]. In Taiwan, herbal products (concentrated scientific herbal granules) covered by the NHI program are manufactured by GMP-certified pharmaceutical companies [22].

The ancient CM literature did not use the word “TB”, but rather considered this pulmonary condition to be related to a syndrome of lung consumption characterized by cough, hemoptysis, tidal fever, night sweats and emaciation due to consumptive or exhaustive overstrain. It was reported that Chinese medicines combined with conventional medicine has beneficial effects for inhibiting Mycobacterium, strengthening the immune system of the body, enhancing the effect of anti-TB drugs, reducing drug resistance, and being relatively safe [23–25]. While several studies have investigated the use of CM in the care of TB patients [23], there is a lack of this kind of survey on the use of CM among patients with a medical history of TB and complicated with long-term respiratory discomforts.

We intended to investigate the utilization of CM among patients with a medical history of TB complicated with long-term respiratory discomforts in this study. The results of this study will be useful for future clinical trials and pharmacological investigations regarding efficacy and safety.

## Materials and methods

### Data sources

The NHI program was established in Taiwan in 1995. The program was highly representative of samples of Taiwan’s general population because the reimbursement policy is universal and mandatory. The coverage rate of this compulsory health insurance program reached almost 95% in 1997 [26] and 99.4% at the end of 2010 in Taiwan [27]. CM services that are reimbursed in the NHI program include Chinese medicines, acupuncture, moxibustion, and Chinese traumatology therapy [28]. The National Health Insurance Administration provided the registration files and original claims data to the National Health Research Institutes, which established and managed the National Health Insurance Research Database (NHIRD) for research purposes. This study was based in part on data from the NHIRD. It contains data comprising demographic characteristics, medical care facilities, outpatient and inpatient visits, visit dates, diagnostic codes, management, prescriptions and medical expenditures. We acquired a randomly selected sample consisting of one million individuals (Longitudinal Health Insurance Database 2000; LHID 2000) from the NHIRD managed and released by the National Health Research Institutes, Taiwan. The diagnostic codes were from the International Classification of Diseases, Ninth Revision, Clinical Modification (ICD-9-CM) formats.

### Ethical consideration

This study followed the ethical standards of the responsible committee and with the Helsinki Declaration of 1964 and later versions. All the datasets were de-identified and encrypted before released by the National Health Research Institutes, Taiwan. All of the individuals or care providers could not be identified in the database. Patient consent was exempted for the total anonymity of all research data in this study. Therefore, the Research Ethics Committee of China Medical University and Hospital approved this study and waived the requirement for informed consent (CMUH104-REC2–115).

### Study population

**Fig. 1:**
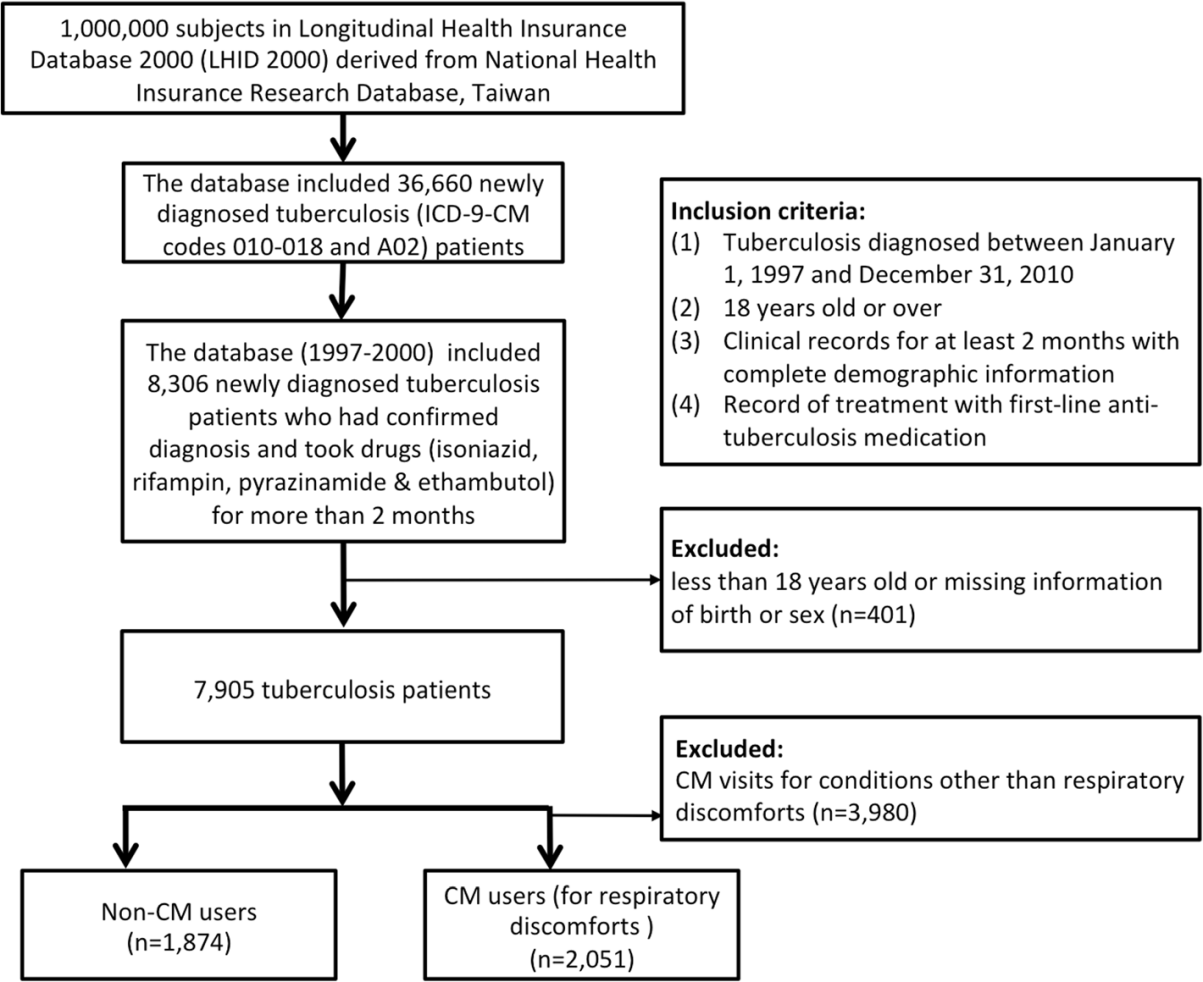
Recruitment flow chart of subjects with tuberculosis (TB) from one million randomly selected samples in the National Health Insurance Research Database (NHIRD) from 1997 to 2000 in Taiwan. The inclusion criteria of patients (*n* = 7905) with history of TB (ICD-9-CM codes 010–018 and A02) were: (1) TB diagnosed between January 1, 1997 and December 31, 2010 (2) 18 years old or over (3) Clinical records for at least 2 months with complete demographic information (4) Record of treatment with first-line TB medication prescriptions. CM users for conditions other than respiratory discomforts (*n* = 3980) were excluded. Finally, a total of 3925 TB patients were categorized as: CM users for respiratory discomforts (*n* = 2051) and non-CM users (*n* = 1874)

Patients newly diagnosed with TB (ICD-9-CM codes 010–018 and A02) (*n* = 36,660) were selected from the database. To avoid the inclusion of patients who did not have the disease, we set the inclusion criteria to allow only patients who were newly diagnosed with TB from January 1, 1997, to December 31, 2010, with clinical records from at least 2 months and treatment with first-line TB medication prescriptions (isoniazid, rifampin, pyrazinamide & ethambutol) (*n* = 8306). Because the prevalence and etiology of TB infections in children were different, and the medical services-seeking behaviors among children or adolescents were mainly dominated by their parents, children with TB infections were not included in our objectives. Therefore, we further excluded patients who were less than 18 years of age or who had missing information related to birth and sex (*n* = 401). A total of 7905 TB patients were then included. Therefore the sample inclusion criteria is as following: (1) TB diagnosed between January 1, 1997 and December 31, 2010. (2) 18 years old or over. (3) Clinical records for at least 2 months with complete demographic information (4) Record of treatment with first-line TB medication prescriptions. CM users were defined as those who had visited CM clinics and had CM outpatient clinical records of TB or respiratory diseases (ICD-9-CM codes 786.x or 460–519). Non-CM users were defined as those who never visited CM clinics after the initial diagnosis of TB. To investigate TB patients complicated with long- term respiratory discomforts, we further excluded those clinical visits with a non-TB or respiratory diagnosis (*n* = 3980). Finally, a total of 3925 TB patients were then categorized into CM users (*n* = 2051) and non-CM users (*n* = 1874) (Fig. 1). Taiwan is a country with a population of 23 million. Taiwan has a geographical area of 36,000 km^2^. There were 23 cities and 359 townships in Taiwan and many of its residents live within urban cities. The residential areas of 23 cities in Taiwan were classified into 4 levels of urbanization based on the population density (people/km^2^), the ratio of the population with varying educational levels, and the number of physicians per 100,000 people. Level 1 represents the highest urbanized level, while 4 represents the lowest level. Levels 1 and 2 of this urbanization were defined as urban areas, while levels 3 and 4 were classified as rural areas [29].

### Prescription of Chinese medicines

We listed the herbal formulas by their phonetic pin-yin name and single herbs by their phonetic pin-yin name, Chinese material medica name and plant name. Indications for the herbal formulas and single herbs were based on CM theory [30, 31]. Full botanical names were in accordance with the International Plant Names List (IPNI; http://www.ipni.org) and The Plant List (http://www.theplantlist.org/) [32]. An open-sourced freeware Node XL (http://nodexl.codeplex.com/) was utilized to identify the core patterns of prescriptions for TB patients, and the most common co-prescribed Chinese medicines were demonstrated in this network analysis. As previously described [33], the larger of the spots with thicker-line widths indicated significant prescription patterns and counts of close connections between formulas and herbs in the network figure.

### Statistical analysis

All statistical analyses were performed using SAS software, version 9.4 (SAS Institute Inc., Cary, NC, USA). Univariate analysis was utilized to compare the CM users with the non-CM users. Data analysis comprised descriptive statistics, including the frequency of herbal prescriptions, patient demographic characteristics, indications for the TCM prescription, and the most frequently prescribed herbal formulas and herbs for treating TB. The chi-square test was used to examine the relationships between the categorical variables and to examine the differences between CM users and non-CM users. A *P*-value of < 0.05 was considered statistically significant.

## Results

In this study, we identified 52.25% (*n* = 2051) patients with a medical history of TB who had visited CM clinics and had CM outpatient clinical records of TB or respiratory diseases (Fig. 1). Those who were female, younger (18–39 y/o), and lived in higher urbanized areas (levels 1 and 2) were relatively more likely to use CM. The median duration between newly diagnosed TB and the first CM consultation was 1068 days (Table 1).

**Table 1:** Demographic characteristics of the patients with tuberculosis in Taiwan

Variable	Non-CM users		CM users^†^		*P*-value
	n = 1874 (44.753%)		n = 2051 (52.25%)		
	n	%	n	%	
Sex					<.0001*
Female	354	18.89	861	41.98	
Male	1520	81.11	1190	58.02	
Age at baseline (year)					<.0001*
18–29	100	5.34	293	14.29	
30–39	127	6.78	242	11.8	
≥ 40	1647	87.89	1516	73.92	
Mean (SD)	64.06 (17.68)		53.25 (18.03)		<.0001‡
Urbanization					<.0001*
1 (highest)	408	21.77	529	25.8	
2	431	23	607	29.61	
3	327	17.45	345	16.83	
4	354	18.89	306	14.93	
5+ (lowest)	354	18.89	263	12.83	
Drug used					
rifampin	1819	97.07	2005	97.76	0.1714*
ethambutol	1796	95.84	1985	96.78	0.116*
isoniazid	1802	96.16	1972	96.15	0.9874*
pyrazinamide	1581	84.36	1798	87.66	0.0028*
levofloxacin	456	24.33	536	26.13	0.1948*
streptomycin	131	6.99	113	5.51	0.055*
kanamycin	73	3.9	81	3.95	0.9308*
prothionamide	18	0.96	24	1.17	0.5237*
cyclosporine	2	0.11	5	0.24	0.456$
moxifloxacin	0	0	1	0.05	–
Interval between the onset oftuberculosis and the first CM consultation, days(median)			1395 (1068)		–

‡ t-test; * chi-square; $ Fisher’s exact test

Abbreviation: *SD* standard deviation, *CM* Chinese Medicine

† CM users referred to patients with history of TB who had visited CM clinics and had CM outpatient clinical records of tuberculosis or respiratory diseases

Regarding the treatment modes employed among the CM users, approximately 95% of CM users (*n* = 1944) received only Chinese medicines, 5.2% of patients received combined treatment with both Chinese medicines and acupuncture, and the remaining 0.2% of patients received only acupuncture treatments. Among all CM users (patients with a medical history of TB who had visited CM clinics and had CM outpatient clinical records of TB or respiratory diseases; *n* = 2051), 90.15% visited CM clinics 1–3 times annually, while 4.58% patients consulted CM doctors more than 6 times/year (Table 2).

**Table 2:** Distribution of CMs according to type of CM treatment received in patients with tuberculosis, stratified by the number of outpatient visits

Number of CM visits (times/per year)	Only Chinese medicines	Only acupuncture	Combination of both treatments	Total *n* = 2051 (100%)
	*n* = 1944 (94.78%)	*n* = 4 (0.20%)	*n* = 103 (5.02%)	
	n (%)	n (%)	n (%)	n (%)
1–3	1763 (90.69)	4 (100)	82 (76.61)	1849 (90.15)
4–6	96 (4.94)	0	12 (11.65)	108 (5.27)
> 6	85 (4.37)	0	9 (8.74)	94 (4.58)

Abbreviation: *CM* Chinese Medicine

The frequency distribution of disease categories related to respiratory discomforts and comorbidities that patients who had been diagnosed TB was analyzed (Table 3). Compared to the non-CM users, the frequencies of outpatient visits among TB patients who used CM were statistically higher across almost all discomforts and comorbidities, except swelling, mass, and lump in chest (Table 3). More than 70% of TB patients used CM because of acute respiratory infections, cough, chronic obstructive pulmonary disease and allied conditions, and other diseases of upper respiratory tract. These results could indicate the comorbidities and complications of TB and may serve as an explanation for why TB patients complicated with long-term respiratory discomforts turn to CM doctors for help (Table 3). We further investigated the prescription pattern of Chinese medicines and identified the ten most commonly prescribed formulas and single herbs. The ten most commonly prescribed single herbs and herbal formulas were analyzed and are listed in Tables 4 and 5, respectively. The most commonly prescribed herbal formulas and single herb were Xiao-Qing-Long-Tang, and Radix Platycodonis (Jie-Geng), respectively.

**Table 3:** Frequency of different diseases related to symptoms of respiratory discomforts or complications in tuberculosis patients

Disease (ICD-9-CM)	Non-CM users		CM users^†^		*P* value*
	n	%	n	%	
Acute respiratory infections (460–466)	1405	74.97	2022	98.59	<.0001
Other diseases of upper respiratory tract (470–478)	564	30.1	1544	75.28	<.0001
Pneumonia and influenza (480–488)	1156	61.69	1393	67.92	<.0001
Chronic obstructive pulmonary disease and allied conditions (490–496)	1307	69.74	1663	81.08	<.0001
Pneumoconioses and other lung diseases due to external agents (500–508)	183	9.77	146	7.12	0.0028
Other diseases of respiratory system (510–519)	952	50.8	853	41.59	<.0001
Dyspnea and respiratory abnormalities (786.0)	313	16.7	488	23.79	<.0001
Stridor (786.1)	25	1.33	124	6.05	<.0001
Cough (786.2)	475	25.35	1519	74.06	<.0001
Hemoptysis (786.3)	211	11.26	375	18.28	<.0001
Abnormal sputum (786.4)	16	0.85	49	2.39	0.0002
Chest pain (786.5)	375	20.01	930	45.34	<.0001
Swelling, mass, or lump in chest (786.6)	32	1.71	50	2.44	0.1101
Hiccough (786.8)	9	0.48	40	1.95	<.0001
Other symptoms involving respiratory system and chest (786.9)	23	1.23	52	2.54	0.0028

* chi-square test

Abbreviation: SD standard deviation, *CM*, Chinese Medicine

†CM users referred to patients with history of TB who had visited CM clinics and had CM outpatient clinical records of tuberculosis or respiratory diseases

**Table 4:** Ten most commonly prescribed herbs for patients with tuberculosis

Pin-yin name	Chinese Materia Medica name	Botanical name	Indication	Number of person-days
Jie-Geng	Radix Platycodonis	*Platycodon grandiflorus* (Jacq.) A.DC	Cough, large amount of sputum, sore throat	18,779
Bei-Mu	Bulbus Fritillariae Thunbergii	*Fritillaria thunbergii* Miq.	Lung heat with thick phlegm, cough due to yin deficiency	18,460
Xing-Ren	Semen Armeniacae	*Prunus armeniaca* L.var. *ansu* Max	Cough with phlegm, cough in older people or weaker bodies	14,610
Yu-Xing- Cao	Herba Houttuyniae	*Houttuynia cordata* Thunb.	Fever, Inflammation of the respiratory tract	8978
Gua-Lou- Ren	Semen Trichosanthis	*Trichosanthes kirilowii* Maxim	Hot cough with sticky phlegm	8905
Gan-Cao	Radix Glycyrrhizae	*Glycyrrhiza uralensis* Fisch	Lung TB, cough with abundance of phlegm, tired and lack of strength, palpitation and short of breath	8054
Huang-Qin	Radix Scutellariae	*Scutellaria baicalensis* Georgi	Cough due to heat syndromes, infection, and hemoptysis	7991
Mai-Men-Dong	Radix Ophiopogonis	*Ophiopogon japonicus* (Thunb.) Ker Gawl.	Cough, weakness, consumption, short of breath, heat from yin deficiency	6934
Dan-Shen	Radix Salviae Miltiorrhizae	*Salvia miltiorrhiza* Bge.	Restlessness, insomnia, irritability, blood deficiency and blood stasis	6508
Wu-Wei- Zi	Fructus Schisandrae	*Schisandra chinensis* (Turcz.) Baill.	Wheezy cough, palpitations, and thirst due to yin deficiency	6154

**Table 5:** Ten most commonly prescribed formulas for patients with tuberculosis

Pin-yin name	Constitutions			Indications in TCM	Number of person- days
	Pin-yin name	Chinese Materia Medica name	Botanical name		
Xiao-Qing-Long-Tang	Ma-Huang	Herba Ephedrae	*Ephedra sinica* Stapf.	Coughing and wheezing with copious, white, stringy sputum that is difficult to expectorate, stifling sensation in the chest, chronic water metabolism problems and thin mucus associated with weakness of lung	16,050
	Gui-Zhi	Ramulus Cinnamomi	*Cinnamomum cassia* Blume		
	Gan-Jiang	Rhizoma Zingiberis	*Zingiber officinale* Rosc		
	Xi-Xin	Herba Asari	*Asarum heterotropoides* F. Schmidt		
	Wu-Wei-Zi	Fructus Schisandrae	*Schisandra chinensis* (Turcz.) Baill.		
	Bai-Shao	Radix Paeoniae Alba	*Paeonia lactiflora* Pall.		
	Ban-Xia	Rhizoma Pinelliae	*Pinellia ternate* (Thunb.) Makino		
	Gan-Cao	Radix Glycyrrhizae	*Glycyrrhiza uralensis* Fisch		
Xin-Yi-Qing-Fei-Tang	Xin-Yi	Magnoliae Flos	*Magnolia biondii* Pamp.	Lung-heat cough with yellow phlegm, and accumulation of lung heat with nasal congestion	15,035
	Huang-Qin	Radix Scutellariae	*Scutellaria baicalensis* Georgi		
	Mai-Men-Dong	Radix Ophiopogonis	*Ophiopogon japonicus* (Thunb.) Ker Gawl.		
	Zhi-Zi	Fructus Gardeniae	*Gardenia jasminoides* J.Ellis		
	Shi- Gao	Gypsum fibrosum	*Hydrous Calcium* Sulfate		
	Zhi-Mu	Rhizoma Anemarrhenae	*Anemarrhena asphodeloides* Bunge		
	Sheng-Ma	Radix Cimicifugae	*Cimicifuga foetida* L. var., intermedia, Regel		
	Bai-He	Bulbus Lilii	*Lilium brownii* F. E. Br.ex Meillez		
	Pi-Pa-Ye	Folium Eriobotryae	*Eriobotrya japonica* (Thunb) Lindl		
	Gan-Cao	Radix Glycyrrhizae	*Glycyrrhiza uralensis* Fisch		
Ma-Xing-Gan-Shi-Tang	Ma-Huang	Herba Ephedrae	*Ephedra sinica* Stapf.	Fever with thirst, wheezing, coughing, labored breathing caused by heat lodged in the lungs where it obstructs the flow of qi	14,185
	Xing-Ren	Semen Armeniacae	*Prunus armeniaca* L.var. *ansu* Maxim		
	Shi- Gao	Gypsum fibrosum	*Hydrous Calcium* Sulfate		
	Gan-Cao	Radix Glycyrrhizae	*Glycyrrhiza uralensis* Fisch		
Ding-Chuan-Tang	Ma-Huang	Herba Ephedrae	*Ephedra sinica* Stapf.	Coughing and wheezing with thick, yellow sputum that is difficult to expectorate. Fever and labored breathing caused by the disrupted flow of lung qi that transforms into heat	10,664
	Xing-Ren	Semen Armeniacae	*Prunus armeniaca* L.var. *ansu* Maxim		
	Ban-Xia	Rhizoma Pinelliae	*Pinellia ternate* (Thunb.) Makino		
	Huang-Qin	Radix Scutellariae	*Scutellaria baicalensis* Georgi		
	Sang-Bai-Pi	Cortex Mori	*Morus alba* L.		
	Bai-Guo	Semen Ginkgo	*Ginkgo biloba* L.		
	Su-Zi	Fructus Perillae	*Perilla frutescens* (L.) Britton.		
	Kuan-Dong-Hua	Flos Farfarae	*Tussilago farfara* L.		
	Gan-Cao	Radix Glycyrrhizae	*Glycyrrhiza uralensis* Fisch		
Yin-Qiao-San	Jin-Yin-Hua	Flos Lonicerae	*Lonicera japonica* Thunb.	Fever, headache, thirst, cough with chills caused by warm pathogens or wind-heat entering the body and attacking the lungs	10,106
	Lian-Qiao	Fructus Forsythiae	*Forsythia suspensa* (Thunb.) Vahl		
	Jie-Geng	Radix Platycodonis	*Platycodon grandiflorus* (Jacq.) A.DC.		
	Niu-Bang Zi	Fructus Arctii	*Arctium lappa* L		
	Bo-He	Herba Menthae haplocalycis	*Mentha haplocalyx* Briq		
	Dan-Dou- Chi	Semen Sojae preparatum	*Glycine max* (L.) Merr.		
	Jing-Jie	Herba Schizonepetae	*Schizonepeta tenuifolia* (Benth.)		
	Dan-Zhu-Ye	Herba Lophatheri	*Lophatherum gracile* Brongn		
	Lu-Gen	Rhizoma Phragmitis recens	*Phragmites communis* Trin		
	Gan-Cao	Radix Glycyrrhizae	*Glycyrrhiza uralensis* Fisch		
Bai-He-Gu Jin-Tang	Shu-Di-Huang	Radix Rehmanniae	*Rehmannia glutinosa* Libosch	Coughing with blood-streaked sputum, wheezing, dry and sore throat, hot palms and soles, night sweats due to yin deficiency of lung and kidney with heat from yin deficiency	9735
	Sheng-Di-Huang	Radix Rehmanniae	*Rehmannia glutinosa* Libosch		
	Mai-Men-Dong	Radix Ophiopogonis	*Ophiopogon japonicus* (Thunb.) Ker Gawl.		
	Bai-He	Bulbus Lilii	*Lilium brownii* F. E. Br.ex Meillez		
	Bai-Shao	Radix Paeoniae Alba	*Paeonia lactiflora* Pall		
	Dang-Gui	Radix Angelicae Sinensis	*Angelica sinensis* (Oliv.) Diels		
	Bei-Mu	Bulbus Fritillariae Thunbergii	*Fritillaria thunbergii* Miq.		
	Jie-Geng	Radix Platycodonis	*Platycodon grandiflorus* (Jacq.) A.DC.		
	Xuan-Shen	Radix Scrophulariae	*Scrophularia ningpoensis* Hemsl.		
	Gan-Cao	Radix Glycyrrhizae	*Glycyrrhiza uralensis* Fisch		
Xing-Su-Yin	Zi-Su	Folium Perillae	*Perilla frutescens* (L.) Britton.	Wheezy cough, nasal congestion, low fever, headache due to a wind-cold pathogen attacking the lung	9343
	Xing-Ren	Semen Armeniacae	*Prunus armeniaca* L.var. *ansu* Maxim		
	Jie-Geng	Radix Platycodonis	P*latycodon grandiflorus* (Jacq.) A.DC.		
	Sang-Bai-Pi	Cortex Mori	*Morus alba* L.		
	Huang-Qin	Radix Scutellariae	*Scutellaria baicalensis* Georgi		
	Mai-Men-Dong	Radix Ophiopogonis	*Ophiopogon japonicus* (Thunb.) Ker Gawl.		
	Bei-Mu	Bulbus Fritillariae Thunbergii	*Fritillaria thunbergii* Miq.		
	Qian-Hu	Radix Peucedani	*Peucedanum decursivum* Maxim		
	Sheng-Jiang	Rhizoma Zingiberis	*Zingiber officinale* Rosc		
	Ju-Hong	Citri Reticulatae Pericarpium Rubrum	*Citrus maxima* (Burm.) Merr.		
	Zhi-Ke	Fructus Citri Aurantii	*Citrus aurantium* L.		
	Gan-Cao	Radix Glycyrrhizae	*Glycyrrhiza uralensis* Fisch		
Zhi-Sou-San	Jie-Geng	Radix Platycodonis	*Platycodon grandiflorus* (Jacq.) A.DC.	Coughing with slight chills and fever, an itchy throat, phlegm that is difficult to expectorate that occurs in externally contracted wind cold	9275
	Jing-Jie	Herba Schizonepetae	*Schizonepeta tenuifolia* (Benth.)		
	Zi-Wan	Asteris Radix	*Aster tataricus* L. f.		
	Bai-Bu	Radix stemonae	*Stemona sessilifolia* (Miq.) Miq.		
	Bai-Qian	Rhizoma Cynanchi Stauntonii	*Cynanchum stauntonii* (Decne.) Schltr.ex H.Lev.		
	Chen-Pi	Pericarpium Citri Reticulatae	*Citrus reticulate* Blanco		
	Gan-Cao	Radix Glycyrrhizae	*Glycyrrhiza uralensis* Fisch		
Cang-Er-San	Cang-Er-Zi	Xanthii Fructus	*Xanthium Sibiricum* Patr. ex Widder.	Nasal obstruction with purulent nasal discharge caused by an external wind pathogen obstructing the protective qi, which is governed by lung, dizziness, frontal headache	9248
	Xin-Yi	Magnoliae Flos	*Magnolia biondii* Pamp.		
	Bo-He	Herba Menthae haplocalycis	*Mentha haplocalyx* Briq		
	Bai-Zhi	Radix Angelicae Dahuricae;	*Angelica dahurica* (Hoffm.) Benth. & Hook.f. ex Franch. & Sav.		
Qing-Zao-Jiu-Fei-Tang	Sang-Ye	Folium Mori	*Morus alba* L.	Wheezing, cough, dry, parched throat, dry nasal passages, headache, fever, irritability, thirst due to invasion of external warm-dryness attacking the lungs and causing damage to the lung qi and yin	8795
	Shi-Gao	Gypsum fibrosum	*Hydrous Calcium* Sulfate		
	Gan-Cao	Radix Glycyrrhizae	*Glycyrrhiza uralensis* Fisch		
	Xing-Ren	Semen Armeniacae	*Prunus armeniaca* L.var. *ansu* Maxim		
	Mai-Men-Dong	Radix Ophiopogonis	*Ophiopogon japonicus* (Thunb.) Ker Gawl.		
	Pi-Pa-Ye	Folium Eriobotryae	*Eriobotrya japonica* (Thunb) Lindl		
	E-Jiao	Colla Corii Asini	*Equus asinus* L.		
	Ren-Shen	Radix Ginseng	*Panax ginseng* C. A. Mey		
	Hu-Ma-Ren	Semen Sesami Nigrum	*Sesamum indicum* L.		

**Fig. 2:**
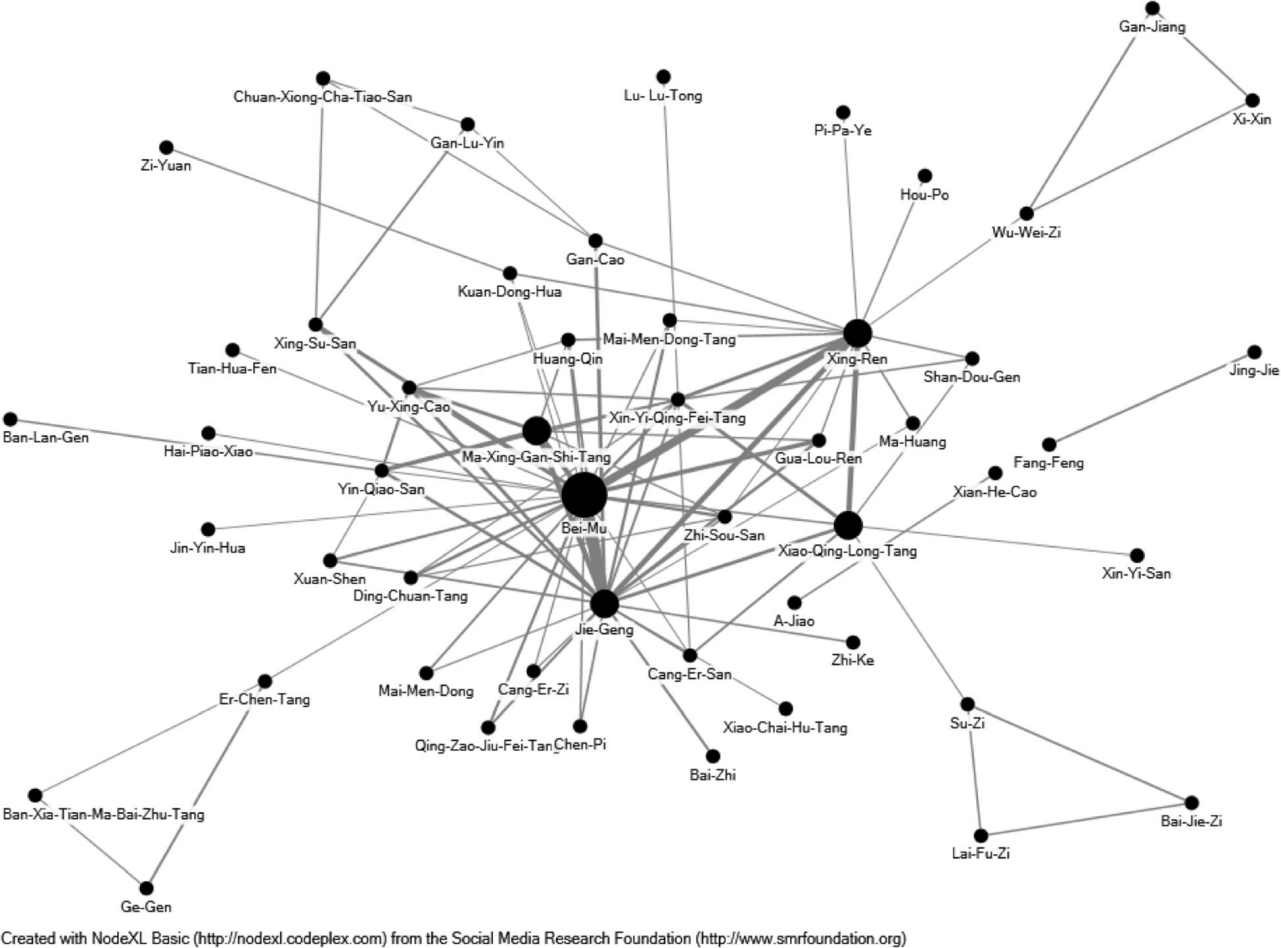
The 100 most commonly prescribed herbal formulas and single herbs for patients with tuberculosis were assessed, and the core pattern of the prescriptions shows that Ma-Xing-Gan-Shi-Tang, Bei-Mu, Jie-Geng, and Xing-Ren were among the most frequently prescribed combinations

**Fig. 3:**
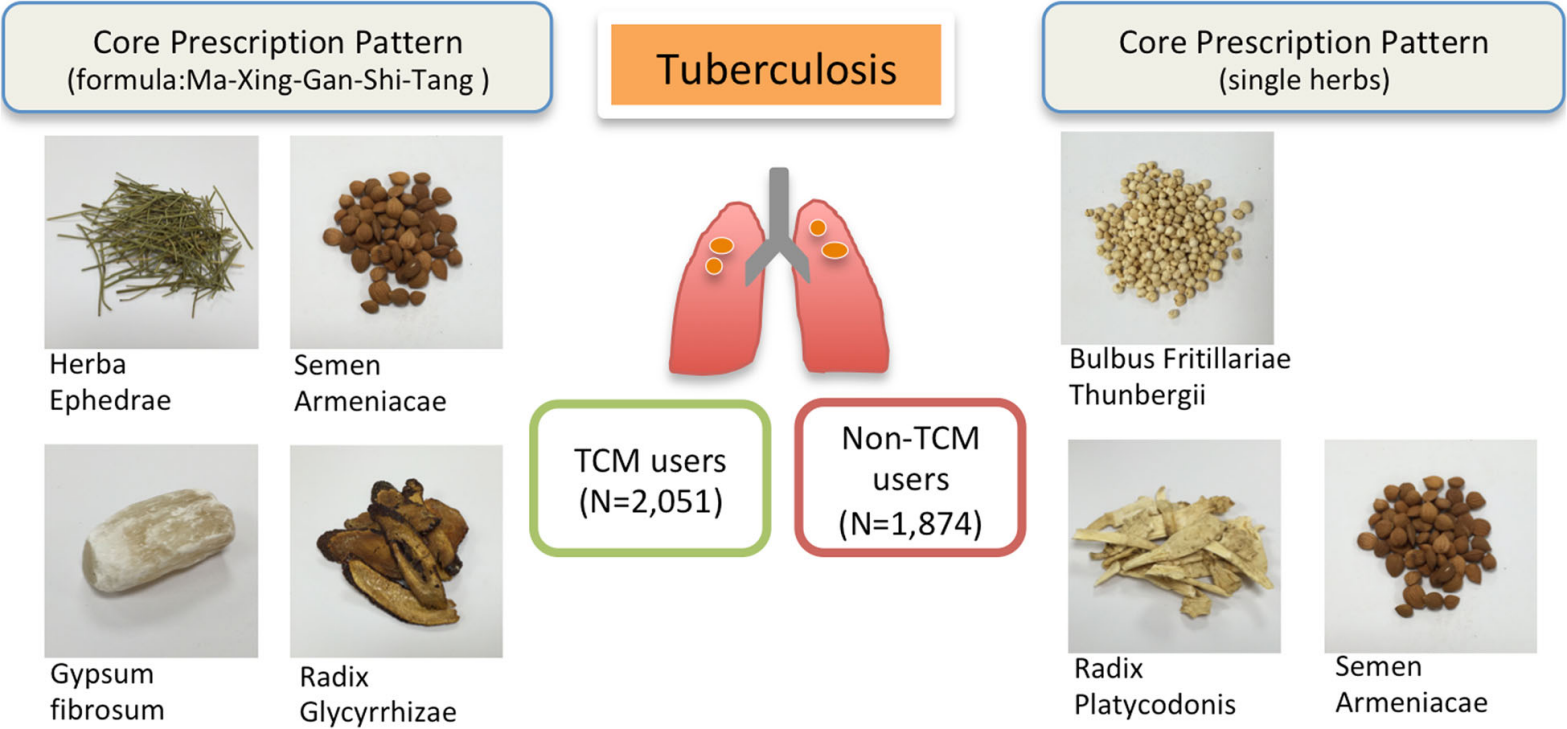
Summarized illustration of the core prescription pattern for patients with tuberculosis in Taiwan

We further conducted a network analysis and found that the core patterns of herbal formulas and herbs prescribed for TB patients consisted of Ma-Xing-Gan-Shi-Tang, Bulbus Fritillariae Thunbergii (Bei-Mu), Radix Platycodonis (Jie-Geng), and Semen Armeniacae (Xing-Ren) (Fig. 2). We further summarized these findings in Fig. 3.

## Discussion

In this study, we first observed that the median duration between newly diagnosed TB and the first CM consultation was 1068 days. Many TB patients complicated with long-term respiratory discomforts sought CM services. We then determined that Ma-Xing-Gan-Shi-Tang, Bulbus Fritillariae Thunbergii (Bei-Mu), Radix Platycodonis (Jie-Geng), and Semen Armeniacae (Xing-Ren) were the core prescriptions for patients with TB in Taiwan. To the best of our knowledge, this report describes the first nationwide population-based cohort study investigating CM utilization patterns among TB patients complicated with long-term respiratory discomforts. However, whether CM combined with modern conventional medicine has beneficial effects or risks requires further investigation.

The phenomenon that female patients with TB were relatively more likely to seek CM services is comparable to a previous Taiwanese survey demonstrating that women had a greater tendency to seek CM consultations [34]. It has been reported that women are more susceptible to side effects from anti-TB drugs due to higher CYP3A activity [11, 35], although men are more susceptible to TB than women [36]. Women may exhibit more help-seeking behaviors than men and are more likely to seek complementary therapies when they suffer from chronic or catastrophic illness [37, 38].

We also observed that younger patients (18–39 y/o) and those who lived in more urbanized areas were relatively more likely to choose CM, in accordance with previous studies [38, 39]. Patients younger than 40 years old are likely to be more aware of alternative therapeutic choices. A previous report had shown that women, people with high socioeconomic status, higher education levels, self-perceived poor health status, and people who exercise regularly tend to visit CM services more often in Taiwan [40]. Patients who reside in more urbanized areas usually have a higher socioeconomic status and fewer barriers to accessing medical services [41, 42]. The time and money spent on transportation may also lead to a significant inequality in contact with the health system [43–45]. Therefore, people who live in urbanized areas have a greater tendency to use CM services.

In Taiwan, patients with a diagnosis of TB are required to receive pharmacological therapy to control TB infection with monitoring by local health officers based on the government’s health policy [46]. The included patients who had been diagnosed with TB should have received standard anti-TB treatment. In Taiwan, recommended standard treatment for adult respiratory TB consists of a regimen of isoniazid, rifampicin, pyrazinamide, and ethambutol for 2 months, followed by isoniazid, rifampicin, and ethambutol for 4 months with monitoring by local health officers according to governmental policy [46]. The total treatment course takes approximately 6 months in newly diagnosed patients [46]. The median duration between newly diagnosed TB and the first CM consultation was 1068 days. During the follow-up period, patients might seek CM consultation due to symptoms of respiratory discomfort or complications. In addition to respiratory illness, they may also experience other symptoms related to the anti-TB treatment, such as skin rash or drug fever, GI intolerance, nausea, vomiting, visual toxicity, hearing disturbances and arthralgia [9, 47–49]. The NHI insurance coverage for CM treatments may play a considerable role in patients’ tendency to seek CM consultations. Medical services in both Western medicine and CM have been promoted due to the high coverage of the NHI program. Furthermore, the cost of co-payment for concentrated scientific herbal granules is approximately $15 U.S. dollars per month under the NHI Program in Taiwan. The tendency of patients to seek CM may continue to increase since the co-payment for Chinese medicines is relatively low [50–53].

Among patients with TB, most CM users (94.78%) received only Chinese medicines, which is consistent with previous studies investigating other respiratory diseases for which herbal products are frequently used as treatment in CM visits in Taiwan [52, 54]. In patients with adult-onset asthma, 76.7% patients who visited CM physicians received Chinese medicines [54], and 97.1% of the CM-treated rhinosinusitis subjects were prescribed Chinese medicines [52].

Regarding the disease categories of the CM users identified in Table 3, most of TB patients used CM because of acute respiratory infections, cough, chronic obstructive pulmonary disease and allied conditions, and other diseases of upper respiratory tract. It was reported that complications of TB include bacterial pneumonia, cor pulmonale, pneumothorax, and acute respiratory failure. Complications of TB could result in progression of functional pulmonary impairment and structural lung destructions [14, 55]. Patients with a prior TB diagnosis were more than twice as likely to have lung airflow obstruction [56]. Furthermore, the complications of respiratory diseases can be independent predictor of shorter survival among patients with TB [57]. TB patients might also turn to CM doctors in the hopes of relieving their symptoms and improving their health [58].

It must be noted that TB did not appear as a disease name in the records of the Chines medical literature. However, ancient people had observed the disease, and the symptoms and signs of TB and its related illness had been recorded and discussed in the Chinese medical literature. The prescriptions of CM were in accordance with the concepts of TB in CM, which were related to chronic cough with symptoms and signs of consumption or exhaustion. The prescriptions were prescribed after pattern identification and syndrome differentiation based on the theory of CM. Most of the prescriptions listed in Tables 4 and 5 are helpful for relieving symptoms caused by yin and qi deficiency of the lung with wind contraction, which corresponds to the respiratory symptoms related to long-term respiratory discomforts [19].

Among the single herbs identified in the core prescriptions, the herb Bulbus Fritillariae Thunbergii has been commonly used as an antitussive agent [59]. Its isosteroidal alkaloids have been reported to have tracheobronchial relaxant activity [60]. Radix Platycodonis has been used for bronchitis, tonsillitis, and laryngitis in ancient times. The pharmacological constituent Platycodin D in Radix Platycodonis has demonstrated anti-inflammatory, antitumor [61], antinociceptive and immunomodulatory activities. Immunostimulatory effects have been revealed via the suppression of IL-6 and TNF-α contents [62] and enhancement of the killing activities of natural killer cells [61]. Radix Platycodonis also inhibits OVA-induced airway inflammation and regulates the production and secretion of airway mucin [63]. Furthermore, Radix Platycodonis has often served as an expectorant in diverse inflammatory pulmonary diseases [64]. The other herb of the core prescriptions, Semen Armeniacae, has anti-inflammatory and analgesic effects by suppressing PGE2 and NO production [65]. The amygdalin found in it has been used as an antitussive, anti-asthmatic, anticancer agent [66, 67].

In addition to the above three herbs, other herbs have been reported to relieve respiratory symptoms or reduce inflammation. Herba Houttuyniae (Yu-Xing- Cao) has been reported to relieve fever, cough and asthma with anti-inflammatory [68] and antiviral activities [69]. This herb inhibits the production of pro-inflammatory cytokines via inhibition of the NF-κB signaling pathway in HMC-1 human mast cells [70]. Semen Trichosanthis (Gua-Lou- Ren) has anti-inflammatory, and antibacterial activities [71] and immunomodulatory effects [72]. Ophiopogonin-D in Radix Ophiopogonis (Mai-Men-Dong) may be beneficial for reducing the excitability of parasympathetic ganglionic neurons in the airways and cholinergic control of airway function, and its antitussive effects might be due to the activation of K+ channels and hyperpolarization of paratracheal neurons [73]. Radix Salviae Miltiorrhizae (Dan-Shen) could be helpful for reducing lung fibrosis [74]. This species has been reported to exhibit anti-inflammatory and antioxidative effects [75]. Radix Scutellariae (Huang-Qin) has shown pharmacological effects that are anti-inflammatory, antitumor and hepato-protective. Additionally, these extracts have antioxidant, antibacterial and antiviral effects [76]. Research has indicated that a decoction containing Fructus Schisandrae (Wu-Wei-Zi) helps to prevent alveolitis and the development of pulmonary fibrosis [77]. Liver-protective effects of Fructus Schisandrae via the inhibitory effect of CYP-3A4 activity have been reported [78] and may also ameliorate the side effects of hepatitis resulting from the use of anti-TB medication.

Among the herbal formulas identified in this research, Ma-Xing-Gan-Shi-Tang is one of the core prescriptions. It has been used to treat asthma [79] and fever associated with pneumonia. It possesses anti-asthmatic, anti-pyretic, anti-inflammatory, and antitussive effects [80, 81]. Xiao-Qing-Long-Tang has anti-inflammatory, antiviral, and anti-allergy activities [82], and it can reduce inflammation in lung tissue via an anti-apoptotic effect and inhibition of cytokine release, as well as prevent pulmonary fibrosis [83–86]. Ding-Chuan-Tang has shown anti-asthmatic effects in clinical studies [87]. Its protective effects against lung injury in asthma occurs by suppressing the production of pro-inflammatory cytokines with significantly reduced inflammatory cell infiltration, goblet cell proliferation, collagen deposition, and damage in the bronchi and alveoli [88]. Xin-Yi-Qing-Fei-Tang has been commonly used for rhinitis [51, 52] and has been shown to reduce eosinophil, serum IgE and IL-4 levels [89]. Clinical studies have reported that Yi-Qiao-San may be helpful for patients with H1N1 influenza to reduce the duration of fever [90]. Lung cancer patients displayed better physical function, role function and cumulative survival after receiving combination treatments of a formula containing Bai-He-Gu Jin-Tang with Western medicine [91].

There are some limitations to our study. First, the herbal products purchased at the patients’ own expense in addition to the NHI program products were not included in this study. However, because the co-payment of herbal products through the NHI program was much less than the market cost, the possibility of purchasing herbs outside the NHI program was relatively low. In addition, compliance to prescription regimens was difficult to measure. Finally, this study focuses on the utilization of CM in patients with TB. In the future, a high-quality randomized controlled clinical trial with imaging data and species cultures to determine the efficacy of Chinese medicines for TB is expected.

## Conclusions

Our study found that many TB patients complicated with long-term respiratory discomforts sought CM services in Taiwan. Those who were 18–39 y/o, female, and who lived in urbanized areas tended to use CM. The prescription patterns identified in this study could be useful for future clinical studies or pharmacological investigations.

## Abbreviations

CM: Chinese Medicine
ICD-9-CM: International Classification of Diseases, Ninth Revision, Clinical Modification
NHI: National Health Insurance
NHIRD: National Health Insurance Research Database
TB: Tuberculosis
